# Treatment updates in myotonic disorders

**DOI:** 10.1007/s00415-026-14073-9

**Published:** 2026-09-04

**Authors:** Emma Matthews, Mark J. Specterman, Karlien Mul

**Affiliations:** 1 Department of Psychology and Neuroscience, School of Health and Medical Sciences, Tooting Campus City St George’s, University of London, Cranmer Terrace, London, UK; 2 Department of Cardiology, School of Health and Medical Sciences, Tooting Campus City St George’s, University of London, Cranmer Terrace, London, UK; 3 Department of Neurology, Donders Institute for Brain, Cognition and Behaviour, Radboud University Medical Centre, Nijmegen, The Netherlands

**Keywords:** Myotonic disorders, Disease modifying, Genomics, Sodium channel blockers, Therapy development

## Abstract

Myotonia is delayed muscle relaxation after forceful contraction. It is due to hyperexcitability of the skeletal muscle membrane. It can arise from primary skeletal muscle ion channel dysfunction, involving chloride or sodium channels, but is also a prominent clinical feature in myotonic dystrophies where altered RNA splicing leads to secondary ion channel dysregulation amongst other systemic manifestations. Clinically, myotonia can range from delayed eye opening to a disabling symptom causing impaired mobility, functional difficulty and sometimes pain. It can also be a “hidden disability” with many patients feeling socially embarrassed by “looking healthy”, yet being unable to do everyday physical tasks or to do them as effortlessly as their peers. It is a symptom that almost always indicates a genetic diagnosis, although it can occur in acquired conditions, including metabolic and drug-induced causes. To experience myotonia without knowing what it is can be baffling. To receive a genetic diagnosis associated with it can be life changing. Although there is no cure, there are many effective and available symptomatic treatments for myotonia and currently we are in an exciting era of clinical trials for new molecular disease-modifying therapies for myotonic dystrophy type 1. In this review, we consider recent developments in the treatment of myotonic disorders and how they may change clinical practice.

## Introduction

Myotonia is delayed muscle relaxation after forceful contraction due to hyperexcitability of the sarcolemma. Myotonia is a prominent symptom of several genetic disorders namely, myotonic dystrophy type 1 and 2 and the non-dystrophic myotonias. In myotonic dystrophies, myotonia occurs in the context of a multisystem disease caused by RNA spliceopathy, whereas non-dystrophic myotonias are primarily skeletal muscle channelopathies affecting chloride or sodium conductance. Myotonic potentials on electromyography, sometimes without significant clinical myotonia, can also indicate Pompe’s disease or be seen in other genetic myopathies with progressive weakness. Very rarely, myotonia can be drug induced. Here, we primarily consider recent updates in clinical therapies and emerging new treatments for the main diagnoses of myotonic dystrophy and non-dystrophic myotonia.

### Myotonic dystrophy type 1

Myotonic dystrophy type 1 (DM1) is the most common myotonic disorder. Prevalence based on clinical diagnosis is estimated between 1 in 6–10 000 [1], while genetic data indicate as many as 1 in 2100 may be affected [2]. DM1 is an autosomal dominant multisystem disorder due to the expansion of an unstable CTG repeat sequence in the 3'-untranslated region of the *DMPK* gene on chromosome 19 [3]. Expanded transcripts result in toxic RNA gain of function, leading to widespread disruption of alternative splicing and explaining the multisystem phenotype of DM1. The clinical features include myotonia, progressive muscle wasting and weakness, cardiac conduction disease, cardiomyopathy, sudden cardiac death, respiratory failure, cataracts, diabetes mellitus, thyroid dysfunction and cognitive dysfunction including learning disability [4]. There is significant variation in clinical presentation, age of onset, and severity and not all patients have all the clinical features. Some patients will live an “almost normal” life, achieve highly in education and work and be unaware they even have the condition until well into adulthood. Others will be significantly disabled, lose ambulation and have shortened life expectancy from cardiorespiratory complications.

### Myotonic dystrophy type 2

Myotonic dystrophy type 2 (DM2) is much less common than type 1. It is due to an expanded CCTG region in the *CNBP* gene on chromosome 3 [5]. It shares a similar RNA-mediated spliceopathy mechanism and its clinical features can be similar to DM1, although the myopathy is proximal rather than distal [6]. Systemic involvement is generally milder in DM2. Key differences are that there appears to be no correlation between repeat length and age of onset or severity, there is no anticipation phenomena, and a congenital form has not been described.

### Non-dystrophic myotonias

The non-dystrophic myotonias are also known as muscle channelopathies. This is because they are caused by variants in sarcolemmal ion channel genes with consequent detrimental impact on channel function and membrane excitability [7]. They are further distinct from myotonic dystrophy in that they are skeletal muscle disorders without systemic involvement and normal life expectancy [8]. Myotonia is the core feature, but episodic weakness may occur and pain and fatigue are prominent symptoms for many.

### Myotonia congenita

Myotonia congenita (MC) is the most common of the non-dystrophic myotonias with a prevalence of around 2–7 in 100 000 [9]. It is due to variants in the *CLCN1* gene which codes for the ClC-1 voltage gated chloride channel essential for muscle membrane repolarisation [10, 11]. This is the same channel whose function is disrupted in myotonic dystrophy causing myotonia. MC can be an autosomal dominant or recessive disorder. Myotonia can affect any skeletal muscle, but typically is most disabling in the leg muscles followed by the hands and eyelids [8]. Myotonia often occurs when movement is attempted after prolonged rest. A warm-up phenomenon is characteristic, i.e. the myotonia becomes less notable with repetitive muscle action.

### Paramyotonia congenita and sodium channel myotonia

These are allelic autosomal dominant disorders, both due to variants in the *SCN4A* gene coding for the voltage-gated sodium channel Nav1.4, essential for action potential generation and muscle contraction [12, 13]. Myotonia is most notable in the muscles of the face and hands followed by legs, but as with MC can be experienced in any skeletal muscle. In paramyotonia congenita (PMC), there is a strong exacerbation of the myotonia with cold temperature and repetitive action. There may also be additional episodes of muscle paralysis, a feature not seen in sodium channel myotonia (SCM). In SCM, individuals can experience myotonia demonstrating both warm-up phenomenon and paradoxical worsening [8].

#### Anti-myotonic treatments

Anti-myotonic treatments can be used in both non-dystrophic myotonias (NDM) and the myotonic dystrophies. The staple approach to symptomatic treatment of myotonia for many years has been to use sodium channel blockers [14, 15]. Sodium channel blockers have a direct mechanism of action in PMC and SCM. This is because *SCN4A* variants have a gain of channel function effect which is inhibited. In myotonia congenita and myotonic dystrophy, their effect is indirect: a reduction in persistent sodium current is thought to partially compensate for impaired chloride-mediated membrane repolarisation.

Functional studies have demonstrated the CLCN-1 gene variants causing myotonia congenita either impair channel gating or reduce expression of the chloride channel at the sarcolemma. No specific chloride channel activators are known, but it has been demonstrated that niflumic acid, a non-steroidal anti-inflammatroy drug, which acts as a chloride channel blocker when applied acutely to transfected HEK cells, conversely acts as a channel chaperone increasing protein expression in vitro when applied over 24 h [16]. Whether the acute channel blockade could be accommodated clinically or overcome by modifying the structure of niflumic acid remains to be seen.

Another off-target approach for pharmacological development in myotonia congenita has been potassium channel openers such as retigabine [15]. Retigabine was permanently withdrawn from the global market in 2017 due to low use and concerns around it causing blue pigmentation, but several next-generation compounds are in development.

### Sodium channel blockers: mexiletine and lamotrigine

Historically, many sodium channel blockers have been used to treat myotonia including phenytoin and carbamazepine, but over time mexiletine has emerged as first-line therapy. This was largely due to the clinical impression of mexiletine as an effective and well-tolerated treatment alongside anecdotal reports.

In 2012, the first randomised controlled trial confirmed this clinical impression, confirming that mexiletine was effective in reducing myotonia severity compared to placebo for NDM, as rated by the interactive voice response (IVR) diaries [17]. Several other studies confirmed this benefit in NDM, which is now well established [18, 19]. The primary outcome in each of the trials was some measure of myotonia, but secondary outcomes consistently showed additional benefits on quality of life, pain, fatigue, activities, independence, social relationships, emotions and well-being.

In 2017, a phase 2 double-blind randomised placebo-controlled cros over trial of lamotrigine provided evidence of efficacy in treating myotonia in 22 patients with NDM based on the myotonia behaviour score (MBS) as the primary outcome [20]. This cemented mexiletine and lamotrigine as the only two treatments for NDM to have RCT evidence of efficacy. A head-to-head study of lamotrigine versus mexiletine failed to demonstrate lamotrigine was non-inferior to mexiletine, but it did still provide considerable efficacy [21].

Both mexiletine and lamotrigine have also demonstrated efficacy in DM1 in terms of improving measures of myotonia [22, 23]. The safety profile including cardiac profile was also favourable, but these studies were relatively small, of short duration and eligibility or selection bias may indicate they do not represent the full population of DM1 in whom cardiac conduction defects are common.

In DM2, evidence for symptomatic anti-myotonic treatment is considerably more limited. Although patients with DM2 have been included in some small studies and observational cohorts, there are currently no dedicated large randomised controlled trials evaluating anti-myotonic therapies in this population, and treatment strategies are largely extrapolated from data in NDM and DM1.

### Cardiac safety considerations for mexiletine and lamotrigine

Mexiletine has attracted some caution in use largely due to its potential pro-arrythmic properties when used in patients with cardiac conduction disease. Although NDMs do not cause cardiac pathology, there has been a conservative approach to cardiac monitoring [24]. Clinical trial and real-world evidence would suggest, however, that this is potentially unnecessary for those with NDM, with none of the trials or 302.4 years of real-world patient follow-up demonstrating any significant ECG changes between baseline and maximal dose [17–19, 25, 26]. The cardiac safety profile of mexiletine in NDM patients appears to be similar to that in the general population [24].

Likewise, both randomised trials and observational studies in DM patients indicate that there is no safety signal for increase in surface ECG cardiac conduction parameters [22, 27, 28]. The patients in those studies tended to have normal cardiac conduction parameters at baseline. In patients with a history of myocardial infarction, all sodium channel blocker risk has been extrapolated from the seminal study of flecainide [29]. However, no formal adverse safety data exists in the literature for mexiletine in this regard and whether this should be extrapolated to all causes of cardiac fibrosis is contentious. Mexiletine appears to be relatively safe, in particular related to cardiac conduction parameters [30, 31]. But with the risk of inherent/developing cardiac conduction disease, serial specialist cardiologist risk-stratified prescribing and monitoring is advisable in DM and particular care in the case of pre-existing conduction disease, fibrosis and/or left ventricular dysfunction [32].

Owing to in vitro data demonstrating class 1B sodium channel blocker activity, the FDA recently issued a warning on the use of lamotrigine in patients with cardiac conduction and/or structural heart disease [33]. Thus far, this has not been replicated by other countries including the European Medicines Agency. It has also not translated to any significant clinical effects on human cardiac conduction in patients with and without pre-existing heart disease or patients with myotonic dystrophy type 1 or NDM [21, 23, 34, 35].

In response, the joint ILAE/AES Task Force issued its own guidance due to the wide use of lamotrigine to treat epilepsy [33]. In brief, it recommends a baseline ECG is only needed for those over 60 years or of any age but with multiple risk factors for cardiovascular disease. In those with known cardiac disease, especially second-/third-degree heart block, Brugada syndrome, arrhythmogenic ventricular cardiomyopathy, left bundle branch block, and right bundle branch block with left anterior or posterior fascicular block, cardiac review and advice must be sought before commencing treatment. At this time, physician discretion is required as to the frequency of ECG monitoring (if at all) when using lamotrigine.

### Lamotrigine or mexiletine?

Mexiletine is licensed for the treatment of adults with NDM under the brand name Namuscla. In several countries, including the Netherlands, generic mexiletine is also routinely used in adults with NDM and is additionally used off-label in children with NDM [36] and in patients with myotonic dystrophy across age groups. The use of lamotrigine as an anti-myotonic drug for any indication remains off-label. Namuscla is considerably more expensive than other options.

A practical limitation of mexiletine therapy is the need for multiple daily dosing, typically up to three times daily, which may affect adherence in some patients. To address this, prolonged-release once-daily formulations of mexiletine are currently being investigated in phase 3 clinical trials involving patients with DM1 and DM2 (HERCULES NCT06523400) and NDM (ACHILLES NCT07097701) [37].

Aside from the cardiac concerns discussed, the risk of other severe adverse drug reactions should also be a factor when considering anti-myotonic treatments. Stevens–Johnson syndrome (SJS) and toxic epidermal necrosis are rare, with an annual incidence of approximately one to two cases per million people, but significant due to high mortality rates. A retrospective 55-year review of the FDA Adverse Event Reporting System Database from 1969 to 2024 found that lamotrigine alone accounted for just under 10% of all cases reported[38]. Comparative factors to consider when offering mexiletine or lamotrigine are summarised in Table 1.

**Table 1:** Comparative factors to consider when prescribing mexiletine or lamotrigine for treatment of myotonia

	Mexiletine	Lamotrigine
Baseline and monitoring ECG required	Yes	No
Cardiac review recommended in patients with history of significant cardiac disease before commencing treatment	Yes	Yes
Safe in pregnancy	Unknown No evidence of teratogenicity Case reports only of use in pregnancy without detriment	Yes Large evidence base to support safety
Time to symptomatic benefit	Rapid—hours to days	Slow—up to weeks
Cost	High	Low
Licensed for treatment of myotonia	NDM adults only	No
Risk of Stevens–Johnson syndrome	Unknown [risk of DRESS (drug reaction with eosinophilia and systemic symptoms) very rare (< 1 in 10 000)]	1 in 1000–2 500
GI side effects	Very common	Very common

#### An era of clinical trials for DM1

Anti-myotonic treatments can be highly effective in treating myotonia. In the case of NDMs, they are the mainstay of treatment as myotonia is the cardinal clinical feature. Myotonic dystrophy is a much more diverse multisystemic disease and treatment requires a multidisciplinary approach [39]. Current management includes symptomatic therapies, including cardiac medication and implantable devices, but no therapies are currently available that modify the progressive muscle weakness and multisystem degeneration characteristic of DM1. This represents a major unmet clinical need. This is now beginning to change with an emerging era of therapeutic clinical trials for DM1 testing new products that target troubling symptoms alongside others that aim to be disease modifying.

### Genetic therapies

**Fig. 1:**
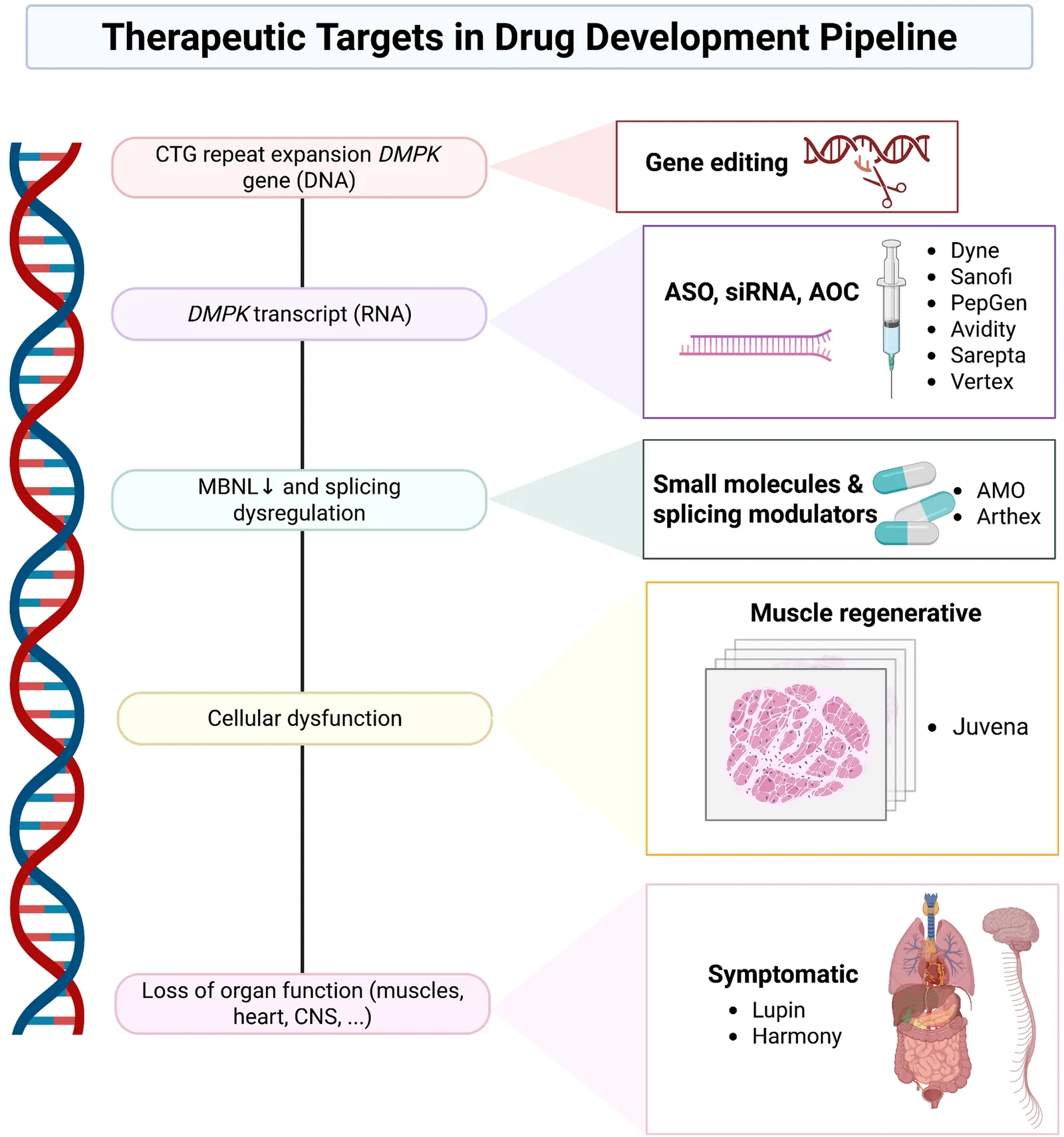
Therapeutic targets in the drug development pipeline

**Fig. 2:**
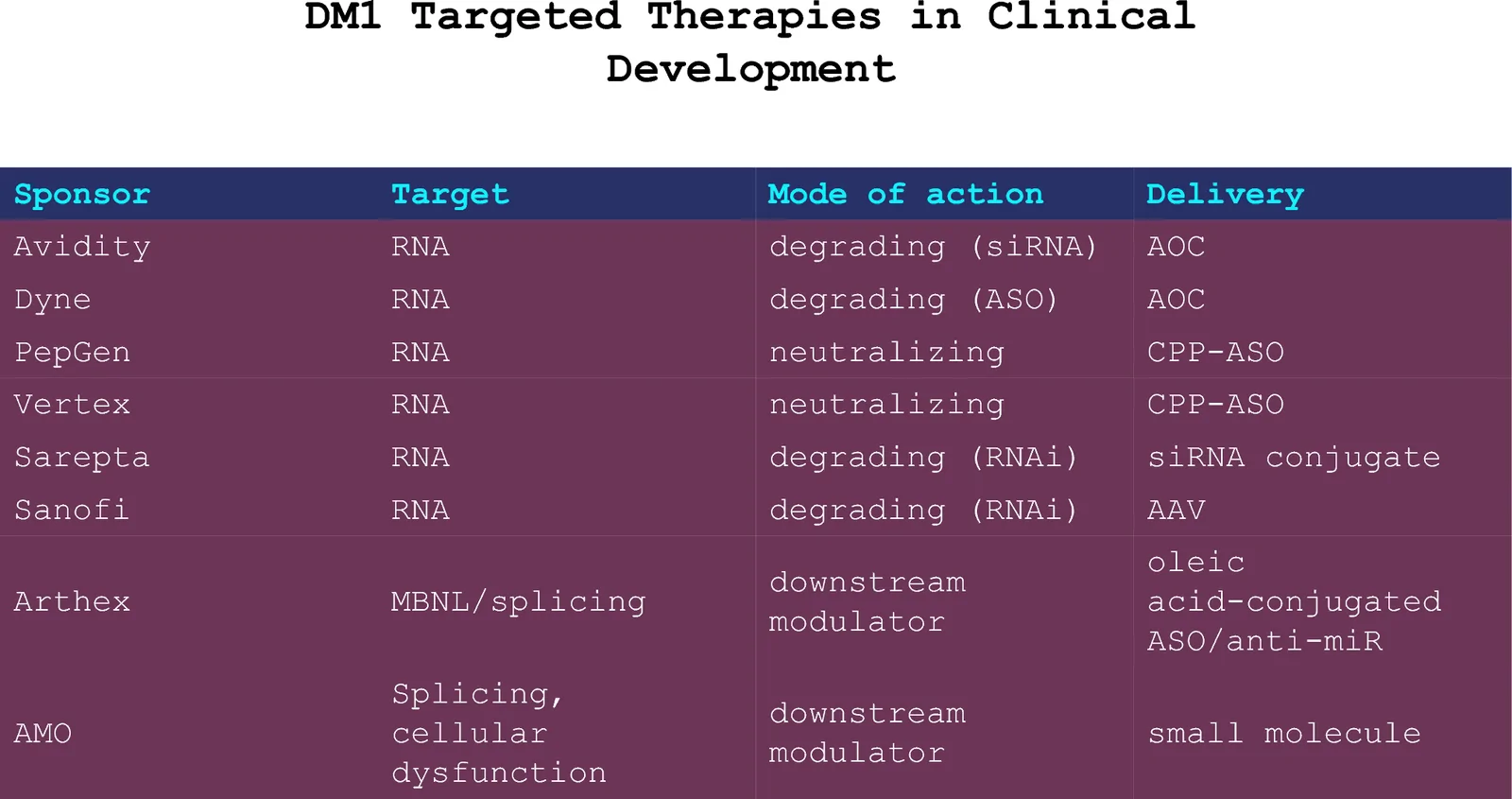
DM1 targeted therapies in clinical development

Several new therapies in development by different commercial companies are targeting and attempting to reduce the mutant *DMPK* transcription or to reduce sequestration of muscleblind-like protein 1 (MBNL1), a key regulator of alternative splicing, with the aim of correcting downstream transcriptional dysregulation (Fig. 1). Different modes of delivery are employed including naked oligonucleotides, targeted oligonucleotides and AAV vectors (Fig. 2). Clinical studies are now in progress across early-phase to phase 3 trials. Most of these new products are being tested in an adult population and exclude those with congenital forms of DM1. The most advanced is an RNA-targeting oligonucleotide approach using an anti–transferrin receptor antibody-oligonucleotide conjugate (del-desiran), which in the phase 2 MARINA trial demonstrated target engagement and amelioration of aberrant alternative splicing in some patients [40].

While these developments are highly promising, they do not represent curative therapies, and the extent of achievable molecular and clinical correction remains uncertain. For most of the trials, the primary outcome measure is myotonia. Many use the video hand opening time (vHOT), an endpoint that remains subject to debate [41, 42]. It captures changes in skeletal muscle excitability and is likely sensitive to modulation of MBNL-dependent mis-splicing of ion channel transcripts, thereby serving as a potential proof-of-mechanism marker. However, it is less clear to what extent improvement in vHOT reflects broader clinical benefit in a multisystem disorder. Whilst other measures of muscle strength and function are employed, the impact on other systems such as cardiac muscle or the brain may not be established for many years and will be dependent on post-marketing surveillance.

A further challenge is delivery: many approaches rely on repeated intravenous administration at 6- to 8-week intervals or complex tissue-targeting strategies, which may limit scalability and CNS penetration, particularly in a multisystem disease such as DM1.

A major consideration for health services will be the practical implementation and affordability should these therapies reach approval. If administered intravenously at 6- to 8-week intervals, this will necessitate substantial healthcare infrastructure and staff resources.

### Small molecules and repurposed therapies

Alongside the development of genetic and RNA-targeted approaches, several repurposed and small-molecule strategies have been explored in DM1, aiming to modulate downstream disease pathways or improve specific functional domains.

Metformin, an anti-diabetic medication with an abundance of historical data on safety and tolerability, has been investigated based on the potential effects on aberrant RNA processing [43]. MYOMET was a single centre, 52-week, double-blind, placebo-controlled phase 2 study, suggesting signal of efficacy in per-protocol analyses using the 6-min walk test (6MWT), although the primary endpoint was not met in the intention-to-treat population [44]. Subsequent studies have suggested that the benefit of metformin may be due to additional mechanisms other than RNA processing [45]. A larger phase 3 trial of metformin in DM1, METFORMYO, is ongoing with the MFM-32 now being the primary outcome measure (NCT05532813).

Tideglusib (AMO-2), a glycogen synthase kinase 3β (GSK3β) inhibitor, has been investigated as a potential disease-modifying approach in DM1. Its rationale is based on evidence that mutant *DMPK* RNA increases GSK3β activity in muscle and the central nervous system, with downstream effects on disease-relevant pathways. After a single blind 2-week placebo period, 16 participants with congenital or childhood DM1 (aged 13–34 years) were randomised to either 400 mg or 1000 mg for 12 weeks of treatment [46]. The investigators were aware of randomisation. There was a reversible and transient increase in liver enzymes in two participants but without clinical correlate and no significant safety concerns arose. Alongside no significant safety concerns, signals of efficacy were seen in cognitive and neuromuscular symptoms as assessed by caregiver- and clinician-reported rating scales, although there was no placebo-controlled arm. A follow-on phase 2/3 study in children and adolescents with congenital DM1 is currently recruiting (NCT03692312).

Other repurposed approaches include macrolide antibiotics such as erythromycin, which have demonstrated preclinical modulation of MBNL-dependent RNA splicing abnormalities [47]. A phase 2 clinical trial showed that erythromycin was well tolerated in DM1 patients and found promising improvements in splicing biomarkers and serum levels of creatine kinase [48].

### Psychostimulants for excessive daytime sleepiness

Excessive daytime sleepiness occurs in the majority of patients with DM1 and can be a major contributor to reduced quality of life. Psychostimulants including modafinil are variably prescribed. Whilst observational studies have suggested benefit, there is very little good quality randomised controlled trial evidence to support this [49].

More recently, a phase 2 placebo-controlled 11-week trial of pitolisant in 30 individuals with DM1 suggested improvements in subjective daytime sleepiness. The study was unfortunately not powered for statistical significance (NCT04886518). Although there was a suggestion of moving to a phase 3 study, this has not yet occurred. Another study of 12 DM1 participants compared variable dose flumazenil with placebo. Whilst use appeared to be safe and tolerated, no benefit was achieved over placebo [50].

## Discussion

The therapeutic landscape of myotonic disorders spans both well-established symptomatic treatment strategies and an emerging pipeline of disease-modifying approaches. For non-dystrophic myotonias, sodium channel blockers such as mexiletine and lamotrigine remain the cornerstone of therapy, supported by randomised controlled trial evidence and long-term clinical experience. In contrast, evidence in myotonic dystrophy is more limited and largely extrapolated, particularly in DM2 where robust trial data are still lacking.

While symptomatic therapies can achieve meaningful improvement in myotonia and related functional outcomes, they do not address the multisystem nature of myotonic dystrophy or its progressive course. This limitation has driven increasing interest in therapies targeting the underlying molecular pathology.

However, the emergence of disease-modifying strategies introduces new translational challenges. In particular, outcome measures such as vHOT predominantly capture skeletal muscle excitability and may not reflect broader disease domains, including cardiac, respiratory and central nervous system involvement. Similarly, heterogeneity in disease expression across DM1 subtypes complicates interpretation of trial results and the generalisability of findings.

Across both symptomatic and disease-modifying domains, an important recurring theme is the gap between physiological or molecular target engagement and clinically meaningful benefit. This applies both to established therapies, where improvements in myotonia do not necessarily translate into broader functional gains, and to emerging genetic approaches, where partial correction of RNA splicing may not equate to reversal of established multisystem disease.

Taken together, the field is characterised not by replacement of symptomatic therapies, but by gradual layering of mechanism-based approaches on top of an existing symptomatic treatment framework. Whether these emerging therapies will ultimately alter disease trajectory remains uncertain, but they represent an important step towards more targeted intervention in myotonic disorders.

Not applicable.
